# Burnout syndrome, doctor-patient relationship and family support of pediatric medical staff during a COVID-19 Local outbreak in Shanghai China: A cross-sectional survey study

**DOI:** 10.3389/fped.2023.1093444

**Published:** 2023-02-13

**Authors:** Baiyu Lyu, Meijia Xu, Lijuan Lu, Xiaoying Zhang

**Affiliations:** Department of Pediatrics, Shanghai Ninth People’s Hospital, Shanghai Jiaotong University School of Medicine, Shanghai, China

**Keywords:** burnout syndrome, pediatric medical staff, COVID-19, doctor-patient relationship, family support, Shanghai comprehensive hospitals

## Abstract

**Objectives:**

To explore burnout syndrome (BOS) incidence, doctor-patient relationship, and family support on pediatric medical staff in Shanghai comprehensive hospitals during a COVID-19 local outbreak.

**Methods:**

A cross-sectional survey of pediatric medical staff from 7 comprehensive hospitals across Shanghai was conducted from March to July 2022. The survey included BOS, doctor-patient relationships, family support, and the related factors of COVID-19. The T-test, variance, the LSD-t test, Pearson's r correlation coefficient, and multiple regression analyses examined the data.

**Results:**

Using Maslach Burnout Inventory-General Survey (MBI-GS), 81.67% of pediatric medical staff had moderate BOS, and 13.75% were severe. The difficult doctor-patient relationship was positively correlated with emotional exhaustion(EE), cynicism(Cy), and negatively with personal accomplishment(PA). When medical staff need help, the greater the support provided by the family, the lower the EE and CY, and the higher the PA.

**Conclusion:**

“In our study, the pediatric medical staff in Shanghai comprehensive hospitals had significant BOS during a COVID-19 local outbreak.” We provided the potential steps that can be taken to reduce the increasing rate of BOS in pandemics. These measures include increased job satisfaction, psychological support, maintaining good health, increased salary, lower intent to leave the profession, regularly carrying out COVID-19 prevention training, improving doctor-patient relations, and strengthening family support.

## Introduction

Burnout syndrome (BOS) is caused by chronic workplace stress that is not adequately addressed (1). Maslach stated that burnout refers to emotional exhaustion, cynicism, and reduced satisfaction of employees in the service industry (2, 3). Emotional exhaustion refers to feeling overextended and depleted, and depersonalization is a sense of cynicism and detached or callous attitude toward aspects of a job. Lack of personal accomplishment refers to perceived incompetence with capabilities in the position (4). The BOS in health care professionals has had significant attention over the last period (5). The American Academy of Pediatrics published a clinical report on doctors' health, acknowledging the high burnout rate of pediatric medical staff, and calling on pediatricians to lead a national campaign to promote the health of medical staff in 2014 (6). Research on BOS among hospital doctors emerged later in China. The research has focused on identifying burnout among comprehensive hospital doctors, the factors contributing to BOS (7), and its impact on medical service delivery (8). BOS has various adverse consequences, including the risk of medical errors, depression, and negative effects on patient safety.

The coronavirus epidemic broke out in Wuhan, China, in December 2019 (9–12). The cause of COVID-19 worldwide led to many illnesses and deaths and the subsequent declaration of a global pandemic (13). During the COVID-19 pandemic, we were exposed to an entirely new, and therefore partially unknown, biological risk (14). Health care institutions are frontline institutions during any disease outbreak or disaster. The COVID-19 pandemic is a global healthcare emergency and economic catastrophe on a scale not seen in more than a century (15). The rapid spread of the pandemic severely tested the health service's ability to respond (14). Frontline healthcare personnel (HCP)are exposed to hazards including pathogen exposure, long working hours, fatigue, burnout, mental health disorders, and physical and psychological violence, potentially negatively impacting patient safety and occupational health (15). With these changes, new stresses arose, some of which differed from previously determined work-related stress factors.

Additional factors associated with high BOS rates included institutional/ departmental elements and increasing clinical demands, such as growing administrative tasks, significant mental health ramifications, and decreased job satisfaction (16). Patients' and doctors' relationships often involve high interpersonal and emotional demands (17, 18). work-home conflicts (8, 19); can also elevate BOS risk. One possible explanation is that the Chinese healthcare service has been rapidly changing over recent years, and a patient-centered approach is gradually replacing the traditional disease-centered care model. Thus, many doctors' responsibilities have increased their patients' demands (19). In addition, the vast population base in China and the corresponding increase in patient numbers have created an enormous work burden for doctors. As a result, doctors work longer hours and have less time to communicate with patients, and medical disputes often occur owing to patient dissatisfaction with care (7). There seemed to be a dialectical relationship between poor doctor-patient relationships and BOS, each contributing to the greater likelihood of the other. Social support refers to the material and spiritual support given to individuals by family, organizations, relatives, colleagues, friends, and partners. It reflects the closeness and quality of a person's social connections (20). Social support can be divided into objective and perceived social support (21). Many studies have also shown a significant negative correlation between social support and BOS (22). Moreover, reports also found that among the three dimensions(family support, friend support, other support) of social support, family support was significantly negatively associated with BOS (22).

Although discussed and reported long before the COVID-19 pandemic, BOS became even more prevalent after its outbreak. Since March 2022, the COVID-19 epidemic situation in Shanghai has been challenging. These challenges affect doctors in all professions; however, the specific problems of the sub-specialty must be considered. The pediatric scale of China's comprehensive hospital is small. The pediatric medical staff needs to undertake the diagnosis and treatment and health care services for children, be responsible for rescuing critically ill neonates in the obstetric department and provide other departments with pediatric consultation tasks. The escalating tension in doctor-patient relationships is a burning issue in China's public healthcare, more prominent in pediatrics (23). However, few studies have investigated the BOS of pediatric medical staff in comprehensive hospitals. Our study aimed to explore BOS incidence and its influencing factors on pediatric medical staff in Shanghai comprehensive hospitals during a COVID-19 local outbreak. So far as we know, this is the first study that evaluates this frequency in Shanghai comprehensive hospitals during the pandemic.

## Materials and methods

### Study design and sampling

The survey was conducted in Baoshan District, Shanghai. In the first stage, medical institutions with pediatric departments were investigated. A total of 7 comprehensive hospitals had pediatric departments. In the second stage, a comprehensive survey was conducted on the on-the-job pediatric medical staff in 7 comprehensive hospitals. A total of 240 medical and nursing staff were given 240 questionnaires, and 240 questionnaires were recovered.

Before carrying out our research, we consulted a focus group composed of qualified pediatricians to identify the profession's specific influencing factors and provide indications for compiling the questionnaire. The questionnaire was composed of 43 questions divided into four parts based on this interview. An online survey form was created using the Wechat questionnaire network. We ensure that the same WeChat account can only answer once. Based on voluntary participation and informed consent, a cross-sectional study was conducted from March to July 2022 to investigate the BOS and its influencing factors on pediatric medical staff in 7 comprehensive hospitals in Baoshan District, Shanghai.

### Questionnaire design

The questionnaire was categorized into four parts.

The first part was some questions about demographic and work-related information. These demographic factors measured age, sex, marital status, health, and educational level. Working factors mainly included hospital grade, job role, working years, professional title and turnover intention.

The second part was a general scale of BOS. The Chinese version of the 15-item Maslach Burnout Inventory-General Survey (MBI-GS) was adopted (24). Including 5 questions on EE, 4 on Cy, and 6 on PA. The internal consistency coefficients (Cronbach's alpha) of EE, Cy and reduced PA are 0.88, 0.83, and 0.82, respectively (24). The questionnaire uses a 7-point Likert scale. With 6 representing “very frequent.” and 0 representing “never.”

The third part was the Doctor-Patient relationship and family support. It has been reported that the high incidence rate of BOS may be directly related to the doctor-patient relationship and family support (25). There seemed to be a dialectical relationship between poor doctor-patient relationships, family support and BOS. The Difficult Doctor-Patient Relationship Questionnaire (DDPRQ) was developed as a valid and efficient method for identifying patients that physicians experience as difficult (26). The DDPRQ-10 requires 10 Likert-type questions with a 6-point response scale, with 1 representing “Not at all “and 6 representing “A great deal. Cronbach's alpha was 0.88 (26). The Perceived Social Support Scale-Family measured family support (25, 27), adapted for research in China by Xiangdong Wang in 1999 (28). This was a questionnaire that consisted of questions in two categories. Responses were scored on a seven-point scale from completely disagree to completely agree. Higher scores indicated more significant levels of perceived support. Cronbach's alpha was 0.869 (25).

The fourth part is the related factors of COVID-19, divided into two sections. The first section concerns pediatric medical staff's psychological problems during COVID-19. The questionnaire includes whether they need psychological support and received psychological support during COVID-19. The second section is about the satisfaction survey during the epidemic. Including satisfaction with work during COVID-19, satisfaction with hospital administration measures, satisfaction with mandatory social distance, and satisfaction with salary. The questionnaire uses a 5-point Likert scale, with 1 representing “very dissatisfied” and 5 meaning “very satisfied.”

### Statistical analysis

SPSS 24.0 software for statistical analysis. Before the data analyses were conducted, the normal distribution of the variables was tested. The P-P-plot and Q-Q-piot analyses of the normal distribution indicated that the variables fulfilled the postulation of normal distribution. Categorical data such as numbers, percentages, and continuous data were presented as means and standard deviations. Independent t-test, analysis of variance and Pearson's r correlation coefficient were used to identify the correlation /association between different demographic characteristics of participants and some influence factors with BOS domains. According to the statistical significance of the results of ANOVA analysis, the LSD-t test performed multiple comparisons between groups. Multiple regression analyses were used to find possible BOS predictors and their three domains. The level of acceptable significance was set at *p* < 0.05.

## Results

### Socio-Demographic data and job characteristics of participants

Two hundred and forty pediatric medical staff participated in this study. 87.92% of respondents were female. Most of them were married. Among the study sample,58.75% were pediatricians and 41.25% were pediatric nurses. 65.83% had completed undergraduate degrees. About half were intermediate titles. Other socio-demographic data and job characteristics of participants are presented in Table 1.

**Table 1:** Socio-demographic data and job characteristics of participants (*n* = 240).

Factors	Categories	*N*	%
Gender	Female	211	87.92
	Male	29	12.08
Age	<30 years	38	15.83
	From 30 to 39 years	99	41.25
	From 40–49 years	72	30.00
	≥50 years	31	12.92
Job role	Pediatrician	141	58.75
	Pediatric nurse	99	41.25
Marital status	Married	199	82.92
	Unmarried	41	17.08
Highest degree attained	Junior college	36	15.00
	Undergraduate	158	65.83
	Master	41	17.08
	Doctorate	5	2.08
Health	Not good	9	3.75
	Commonly	90	37.50
	Good	131	54.58
	Very healthy	10	4.17
Years of work	<5 years	27	11.25
	From 5 to 9 years	44	18.33
	From 10 to 19 years	84	35.00
	≥20 years	85	35.42
Hospital-grade	Small medical institutions	53	22.08
	Secondary hospital	72	30.00
	Tertiary hospital	115	47.92
Professional title	Primary	80	33.33
	Intermediate	133	55.42
	Advanced	27	11.25
Turnover intention	YES	39	16.25
	NO	201	83.75
Do you need psychological support during COVID-19?	YES	148	61.67
	NO	92	38.33
Did you get any psychological support during COVID-19?	YES	56	23.33
	NO	184	76.67

### Prevalence of BOS and the degree of severity of affection within each domain

A total EE score greater than 14 indicates a high degree of EE, while a total score of EE less than 11 indicates a low degree (29). A total score of Cy above 7 indicates a high degree of Cy, while a total score below 5 indicates a low degree (29). A score of over 29 in PA indicates a low sense of achievement, while a score of less than 26 indicates a high sense of achievement (29). The total score of BOS = 0.4 × EE average + 0.3 × Cy average + 0.3 × PA average. Total scores that fall into 0 ∼ 1.49, 1.50 ∼ 3.49, and 3.50 ∼ 6 indicate the conditions of no BOS, moderate BOS, and severe BOS, respectively (30). The prevalence of BOS among the sample was 95.42%, of which 81.67% were moderate burnout. The severity of different domains of BOS are listed in Table 2.

**Table 2:** Severity of BOS domains among participants (n = 240).

Degree of job burnout	** **	*N*	%	Mean	SD
EE	Low (≤10)	119	49.58	6.80	2.39
	Medium (11–14)	51	21.25	12.20	1.00
	High (≥15)	70	29.17	19.13	4.31
Cy	Low (≤4)	93	38.75	2.69	1.59
	Medium (5–7)	47	19.58	5.94	0.84
	High (≥8)	100	41.67	12.55	4.03
PA	Low (≥30)	49	20.42	32.59	2.40
	Medium (26–29)	25	10.42	27.32	1.22
	High (≤25)	166	69.17	18.46	4.33
Total (=0.4*EE + 0.3*Cy + 0.3*PA)	No (≤1.49)	11	4.58	1.17	0.29
	Moderate (1.5–3.49)	196	81.67	2.42	0.48
	Severe (≥3.5)	33	13.75	4.11	0.58

EE, Emotional exhaustion; Cy, Cynicism; PA, Personal accomplishment.

### Association between demographic characteristics and related factors with the three BOS domains

Results suggest that participants' socio-demographic characteristics influenced the degree of BOS. Higher EE scores were significantly associated with age. The pediatric medical staff with working-age >20 years had the most significant EE. EE and Cy are the most significant when health is not good. The EE scores of secondary hospitals and advanced professional titles increased significantly. EE and Cy increased significantly when there was turnover, needed psychological support, and did not get psychological help. The average score of personal achievement of different factors is less than 25 points, except that the average score of excellent health is 27.10, indicating that personal achievement is high, and there is no statistical difference. The results are shown in Table 3. According to the statistical significance of the results of ANOVA analysis, the LSD-t test performed multiple comparisons between groups. The results showed that: (1) Age: <30 years, compared with 40–50 years and >50 years, EE score decreased significantly (*P* < 0.05); from 30 to 40 years compared with 40–50 years and >50 years, EE score decreased significantly(*P* < 0.05). (2) Years of work: >20 years compared with other groups, EE score increased significantly (*P* < 0.05). (3) Health: not good compared with good and excellent, EE and Cy score increased significantly(*P* < 0.05); commonly compared with good and excellent, EE and Cy score increased significantly (*P* < 0.05); good compared with excellent, increased (*P* < 0.05). (4) Hospital- grade significantly: small medical institutions compared with secondary and tertiary hospitals, EE score decreased significantly (*P* < 0.05). (5) Professional title: primary, intermediate compared with advanced, score decreased significantly(*P* < 0.05).

**Table 3:** Association between demographic characteristics and related factors with different BOS domains.

Factors	Categories	EE			Cy			PA		
		Mean	SD	*p*-value	Mean	SD	*p*-value	Mean	SD	*p*-value
Gender^a^	Female	11.36	6.00	0.24	7.35	5.25	0.54	22.35	6.86	0.66
	Male	12.86	6.36		8.03	5.62		21.66	7.99	
Job role^a^	Pediatrician	12.03	5.46	0.14	7.61	5.00	0.55	22.50	7.06	0.54
	Pediatric nurse	10.85	6.76		7.18	5.70		21.94	6.92	
Age^b^	<30 years	10.11	5.12	0.01	6.29	4.19	0.36	20.76	8.00	0.23
	From 30 to 40 years	10.59	5.60		7.41	5.30		22.32	6.91	
	From 40 to 50 years	12.47	6.58		7.58	5.56		22.13	6.33	
	>50 years	14.19	6.25		8.55	5.78		24.26	7.23	
Years of work^b^	<5 years	10.70	5.28	0.01	6.93	4.91	0.60	20.00	6.96	0.21
	From 5 to 10 years	9.98	5.15		6.82	4.99		21.66	7.87	
	From 10 to 20 years	10.79	5.43		7.33	4.86		22.42	6.47	
	>20 years	13.36	6.87		8.01	5.96		23.15	6.95	
Marital status^a^	Married	11.60	6.22	0.70	7.56	5.33	0.39	22.06	6.80	0.35
	Unmarried	11.24	5.18		6.80	5.10		23.29	7.85	
Highest degree attained^b^	MBBCH	10.28	7.06	0.55	6.42	5.51	0.21	22.81	6.96	0.25
	Diploma	11.85	6.09		7.75	5.45		22.49	7.06	
	Master	11.34	5.06		7.56	4.44		20.51	6.55	
	Doctorate	12.40	4.04		3.60	3.36		25.60	7.57	
Health^b^	Not good	16.11	5.62	<0.001	10.78	5.61	<0.001	21.78	5.45	0.17
	Commonly	13.27	6.38		8.89	5.40		22.06	6.60	
	Good	10.43	5,47		6.43	4.90		22.08	7.33	
	Excellent	6.50	3.44		4.40	4.76		27.10	6.99	
Hospital-grade^b^	Small medical institutions	9.09	3.93	<0.001	6.45	4.02	0.28	22.51	6.24	0.49
	Secondary hospital	13.01	6.84		7.96	5.76		22.94	7.00	
	Tertiary hospital	11.75	6.02		7.55	5.48		21.73	7.32	
Professional title^b^	Primary	10.58	5.56	0.01	7.14	4.89	0.56	21.78	6.92	0.20
	Intermediate	11.51	6.04		7.41	5.27		22.11	7.11	
	Advanced	14.56	6.68		8.41	6.48		24.52	6.41	
Turnover intention^a^	YES	16.82	6.07	<0.001	13.23	4.69	<0.001	19.79	6.59	0.20
	NO	10.52	5.50		6.31	4.63		22.75	6.98	
Do you need psychological support during COVID-19?^a^	YES	12.54	6.16	<0.001	8.41	5.53	<0.001	21.81	6.78	0.21
	NO	9.93	5.52		5.87	4.48		23.00	7.29	
Did you get any psychological help during COVID-19?^a^	YES	9.02	5.16	<0.001	5.38	4.58	<0.001	23.63	6.66	0.09
	NO	12.31	6.10		8.06	5.34		21.85	7.05	

EE, Emotional exhaustion; Cy, Cynicism; PA, Personal accomplishment.

^a^ Independent *t*-test.

^b^ Analysis of variance (ANOVA).

### Correlation analysis between related factors with the three BOS domains

Table 4 shows that job satisfaction, forced social distance and COVID-19 administration are negatively correlated with EE, Cy, and positively correlated with PA. The more satisfied the salary, the lower the EE and Cy. The difficult doctor-patient relationship was positively correlated with EE, Cy and negatively with PA. When medical staff need help, the greater the support provided by the family, the lower the EE and Cy, and the higher the PA.

**Table 4:** Correlation analysis between related factors and BOS.

Factors	EE		Cy		PA	
	r	*p*-value	r	*p*-value	r	*p*-value
Job Satisfaction	−0.47	<0.001	−0.45	<0.001	0.15	<0.001
Satisfaction with forced social distance	−0.33	<0.001	−0.38	<0.001	0.20	<0.001
Satisfaction with COVID-19 administration	−0.40	<0.001	−0.40	<0.001	0.16	<0.001
Satisfaction with salary	−0.46	<0.001	−0.41	<0.001	0.10	0.06
Difficult Doctor-Patient Relationship	0.350	<0.001	0.53	<0.001	−0.27	<0.001
Family support	−0.32	<0.001	−0.39	<0.001	0.19	<0.001

EE, Emotional exhaustion; Cy, Cynicism; PA, Personal accomplishment; r, denote the direction of the relationship.

### Predictors of BOS among participants

The multiple regression analysis showed significant differences among BOS variables, with the three dimensions of BOS as three independent variables. EE (F 15.059, R2 0.502), Cy (F 14.462, R2 0.492), PA (F 1.993, R2 0.118). The COVID-19 pandemic added new factors to the development of BOS in this research. We found that education is an independent influencing factor of BOS. The worse the health status, the more obvious the BOS. In addition, salary satisfaction, satisfaction with administrative work, turnover intention, and psychological support during the epidemic of COVID-19, family support were also factors for the development of BOS.as shown in Table 5.

**Table 5:** Multiple regression analysis of the factors associated with BOS.

Variables	EE				Cy				PA			
	B	standard error	Beta	*p*-value	B	standard error	Beta	*p*-value	B	standard error	Beta	*p*-value
Age	0.516	0.614	0.077	0.402	0.402	0.543	0.069	0.460	0.143	0.945	0.019	0.880
Years of work	−0.235	0.588	−0.039	0.689	−0.479	0.519	−0.090	0.357	1.016	0.904	0.144	0.263
Highest degree attained	−1.663	0.534	−0.174	0.002	−1.487	0.472	−0.178	0.002	0.051	0.822	0.005	0.950
Health	−1.090	0.483	−0.114	0.025	−0.715	0.427	−0.086	0.096	0.193	0.744	0.018	0.795
Hospital-grade	0.559	0.387	0.074	0.150	−0.081	0.342	−0.012	0.814	0.039	0.596	0.004	0.947
Professional title	1.008	0.643	0.105	0.119	0.503	0.569	0.060	0.378	−0.015	0.990	−0.001	0.988
Job Satisfaction	−1.088	0.729	−0.125	0.137	−0.264	0.644	−0.035	0.682	−0.404	1.122	−0.040	0.719
Satisfaction with forced social distance	0.225	0.614	0.026	0.714	−0.628	0.543	−0.082	0.248	1.537	0.945	0.153	0.105
Satisfaction with COVID-19 administration	−1.181	0.566	−0.142	0.038	−1.086	0.500	−0.149	0.031	1.360	0.871	0.141	0.120
Satisfaction with salary	−1.887	0.391	−0.303	<0.001	−1.155	0.345	−0.212	0.001	−0.152	0.601	−0.021	0.800
Turnover intention	−2.476	0.863	−0.151	0.005	−4.115	0.763	−0.288	<0.001	1.166	1.328	0.062	0.381
Do you need psychological support during COVID-19?	−1.717	0.628	−0.138	0.007	−1.306	0.555	−0.120	0.019	0.262	0.967	0.018	0.787
Did you get any psychological help during COVID-19?	1.276	0.755	0.089	0.093	1.045	0.667	0.084	0.119	−0.686	1.162	−0.042	0.555
Difficult Doctor-Patient Relationship	0.096	0.052	0.052	0.065	0.005	0.046	0.005	0.915	0.045	0.080	0.037	0.573
Family support	−0.153	0.147	−0.057	0.299	−0.304	0.130	−0.130	0.020	0.261	0.226	0.084	0.250

EE, Emotional exhaustion; Cy, Cynicism; PA, Personal accomplishment.

## Discussion

Herbert Freudenberger first described burnout in 1974. BOS is a state of physical and mental exhaustion related to work or caregiving activities (31). With prevalence near or exceeding 50%, BOS has become a severe mental health problem for healthcare professionals (32, 33). In 2019, the World Health Organization (WHO) recommended for the first time that “burnout” should be included in the International Classification of Disease 11th Edition (34). Furthermore, the COVID-19 pandemic has caused a growing burden on medical services in developing and developed countries. During the COVID-19 pandemic, many studies showed that the stress and burnout of medical staff were widespread, which affected their quality of life and the quality of health services provided (11). Prolonged wearing of personal protective equipment, sleep deprivation, and alimentation accentuate fatigue and burnout syndrome (5). Another apparent source of stress is the increased risk of exposure to COVID-19 among these pediatric medical staff, raising worries about contracting the disease and/or transmitting it to their families and loved ones (1). Some studies in China and UK have also revealed depression and anxiety due to the COVID-19 outbreak that medical staff experienced (35–37).

Research in China during the COVID-19 pandemic found that the proportion of depression, anxiety, and alcohol consumption was higher than usual, and the level of mental health was low (10, 38). Many studies conducted that around one-third of the Chinese population suffered from various forms of anxiety and depression, and one-third of people reported lower mental well-being (10, 38–41). Since March 2022, the COVID-19 epidemic in Shanghai has been challenging; the number of newly infected people has reached tens of thousands daily. Using MBI-GS, 81.67% of pediatric medical staff had moderate BOS, and 13.75% were severe. This indicates that the BOS of medical staff has become a common phenomenon in China and has needed widespread attention. In this study, 87.92% of respondents were female. The great majority of them were married and had children. As everyone knows, women are the majority of primary caregivers, spend more time on parenting, and provide more childcare and domestic tasks than their male counterparts (13, 42). Recent studies have also highlighted the disparity in clinical and academic productivity between women and men to the more outstanding non-work-related obligations women face (13). There is a severe shortage of pediatric medical staff in China, so the workload of these professionals is heavy, which will lead to emotional exhaustion in the long run. The medical environment is often related to the hospital level and the number and quality of patients. The COVID-19 pandemic has left pediatric medical staff with new challenges. The loss of security highlight the urgent need for effective and present leadership to help staff process and handle these challenges. It is essential to continue open communication with opportunities for faculty and staff input and feedback (43).

The higher the professional title, the greater the responsibility. Because leaders with higher professional titles must simultaneously be competent in many tasks, such as clinical diagnosis and treatment, scientific research, and epidemic prevention. Therefore, the higher the professional title, the more serious the emotional exhaustion. In China, doctors have been confronted with many challenges for years. On the one hand, there is a severe imbalance between doctors' heavy workload and low income. On the other hand, many violent incidents against medical staff have risen (44, 45). These phenomena are more evident among pediatric medical staff, making pediatric medical staff an increased incidence of turnover. During the COVID-19 outbreak, questions arise around the equipoise between a physician's duty to care for patients, their obligations to protect their families, and their right to defend their health (15). Notable, medical staff were often overwhelmed with mental health status disorders which may, in turn, compromise patient safety (15). Medical staff providing care to COVID-19 patients are more severely affected by mental health status disorders associated with depression, anxiety, distress and insomnia, stress, and indirect traumatization than other occupational groups (15, 46, 47). The new working conditions period the pandemic have exacerbated compassion fatigue (48, 49), associated with contact with relatives at the end of life and therapeutic failure (50, 51). Organizational measures are vital to support nurses’ medical staff's mental health and address their fear of COVID-19 through social and peer support, psychological and mental support services, and training related to COVID-19 accurate and regular information updates (52).

The rapid spread of the pandemic has seriously tested the response-ability of health service departments. Hence, the COVID-19 pandemic led to a certain amount of logistic and therapeutic uncertainty that required close monitoring and well-trained personnel (53). During this time of mandated social distancing, departments and institutions should make timely adjustments, given the wide-ranging uncertainty regarding the virus and its implications (54, 55). Practice changes included ensuring safe work environments to protect staff best while providing good patient care. An important measure in ensuring safety was securing adequate personal protective equipment (56). Prior to the pandemic, one crucial driver of BOS was a lack of effective leadership, communication and transparency (57). Most survey respondents endorsed departmental leadership updates during the pandemic, communicated frequently, and felt comfortable contacting department leadership regarding related concerns or anxieties.

The escalating tension in doctor-patient relationships is a burning issue in China's public healthcare and has received increasing attention (58). In the past 20 years, the incidence and severity of medical disputes have significantly increased (44). One fundamental reason is that hospital services are not effectively differentiated into hospital care and primary care (59). Another crucial reason patients distrust their doctors lies in the information asymmetry between patients and doctors (60). Furthermore, poor doctor-patient relationships in China can also be attributed to patients' excessive expectations. Their relationship will deteriorate dramatically when patients pay an overwhelming bill without being cured or when medical incidents occur (61). Poor investment in hospitals from the government, media sensationalism, flawed healthcare system, marketization reform of healthcare, and the unethical conduct of medical staff are considered the major factors leading to the issue (62). The poor doctor-patient relationship increases the BOS of the pediatric medical team. The smaller the family support, the more significant the emotional exhaustion, cynicism and low sense of achievement.

There are some limitations of our study. First, the sample size is relatively small. There are many hospitals in Shanghai. The participants in this questionnaire are mainly concentrated in the Baoshan District of Shanghai, lacking the representation of pediatric medical staff in other regions; Secondly, as a cross-sectional study, the research captured the situation of BOS at a single time point. There is no comparison of pre-COVID-19 data to reflect the impact of COVID-19 on pediatric health worker burnout. Only the investigation conducted in a more extended period and potential longitudinal research in future research will supply us with a better knowledge of BOS; Finally, in the design of the satisfaction questionnaire, the relationship between medical staff's mental health problems (such as insomnia, anxiety, and depression) and BOS during the COVID-19 epidemic should be paid more attention to. However, there are still deficiencies in this study's design of mental health problems.

## Conclusions

The COVID-19 pandemic has raised public health problems worldwide and required a reorganization of health services. In this context, the severe BOS of pediatric medical staff in Shanghai comprehensive hospitals during a COVID-19 local outbreak. The government and hospital managers should take targeted measures. First of all, we should enhance the job attraction of pediatric medical staff, increase the source of human supply and improve the economic return. Secondly, optimize the proper flow of personnel within the Department and alleviate the work intensity of clinicians. Moreover, we can improve the medical staff's correct understanding of COVID-19 through training and effective psychological counseling. Finally, the harmonious pediatric doctor-patient relationship is based on perfect hospital and department management mechanisms, excellent professional medical staff, and mutual understanding and trust in underdiagnosis and treatment.

## Data Availability

The raw data supporting the conclusions of this article will be made available by the authors, without undue reservation.
