# Supplementary material for: Burnout syndrome, doctor-patient relationship and family support of pediatric medical staff during a COVID-19 Local outbreak in Shanghai China: A cross-sectional survey study
Source: Front Pediatr. 2023 Feb 13;11:1093444. doi: 10.3389/fped.2023.1093444 (PMC9968926; doi:10.3389/fped.2023.1093444)
Supplement: Supplementary file 1 [file Datasheet1.pdf]

Questionnaire design

The first part: demographic and work-related information. (10 items)

Q1_ What is your occupation?

1 pediatrician, 2 pediatric nurse

Q2_ What's your gender?

1 female,2 male

Q3_ What's your age?

1 < 30y,2 30-40y, 3 40-50y,4 > 50y

Q4_ What is your length of service?

1 < 5y ,2 5-10y ,3 10-20y, 4 > 20y

Q5_ Marital status?

1 Married,2 Unmarried

Q6_ Highest degree attained?

1 Associate,2 Bachelor,3 Master,4 Doctorate

Q7_ What is your health status?

1 Not good,2 Commonly,3 Good,4 Very healthy

Q8_ What is your hospital level?

1 Small medical institution,2 Secondary hospitals,3 Tertiary hospitals

Q9_ What is your professional title?

1 Primary,2 Intermediate,3 Advanced

Q10_ Do you have any intention to leave?

1 Yes,2 No

The second part: the Chinese version of the 15-item Maslach Burnout Service Inventory (MBI-GS). 0 never, 1 very few times (several times a year or less), 2 a few times (once a month or less), 3 a little more (several times a month), 4 a few times (once a month or less), 5 very few times (several times a year or less), 6 very frequent(per day) (15 items)

Emotional exhaustion(5)

Q11_ work makes me feel physically and mentally exhausted.

Q12_ I feel exhausted when I get off work.

Q13_ When I got up in the morning and had to face the day's work, I felt exhausted.

Q14_ Working all day is stressful for me.

Q15_ work makes me feel like I'm going to collapse.

Cynicism(4)

Q16_ Since I started this job, I have become less and less interested in it

Q17_ I'm not as enthusiastic about my work as I used to be

Q18_ I doubt the significance of my work

Q19_ I'm becoming less and less concerned about my contribution to my work

Personal accomplishment(6)

Q20_ I can effectively solve problems in my work

Q21_ I think I'm making a valuable contribution to the unit

Q22_ In my opinion, I am good at my work

Q23_ I am pleased when I finish some things in my work
Q24_ I have done a lot of valuable work
Q25_ I am confident that I can finish all kinds of work effectively

The third part was the Doctor-Patient relationship and family support. （10 items）
The Difficult Doctor-Patient Relationship Questionnaire-10
1 - "Not at all," 6 = "A great deal.
Q26_How much are you looking forward to this patient's next visit after seeing this patient today?
Q27_ How "frustrating" do you find this patient?
Q28_ How manipulative is this patient?
Q29_ How difficult is it to communicate with this patient?
Q30_ To what extent are you frustrated by this patient's vague complaints?
Q31_ How self-destructive is this patient?
Q32_ Do you find yourself secretly hoping that this patient will not return?
Q33_ How at ease did you feel with this patient today?
Q34_ How time-consuming is caring for this patient?
Q35_ How enthusiastic do you feel about caring for this patient?

Family support(2). Responses were scored on a seven-point scale from completely disagree to completely agree. （2 items）
Q36_My family can help me concretely; I can obtain emotional help and support from my family when I need it
Q37_I can talk to my family about my problems.

The fourth part is the related factors of COVID-19. （2 items）
Q38_ Do you need psychological support during COVID-19?
1 Yes,2 No
Q39_ Did you get any psychological support during COVID-19?
1 Yes,2 No
The questionnaire uses a 5-point Likert scale, with 1 representing " very dissatisfied" and 5 meaning "very satisfied. （4 items）
Q40_Satisfaction with work during COVID-19
Q41_ Satisfaction with hospital administration measures during COVID-19
Q42-Satisfaction with mandatory social distance
Q43- Satisfaction with salary
